# Two-dimensional transthoracic measure of mitral annulus in mitral valve prolapse and moderate to severe regurgitation: a method comparison analysis with three-dimensional transesophageal echocardiography

**DOI:** 10.1186/s44348-024-00001-w

**Published:** 2024-06-12

**Authors:** Maxime Berthelot-Richer, Halyna Viktorivna Vakulenko, Anna Calleja, Anna Woo, Paaladinesh Thavendiranathan, Frédéric Poulin

**Affiliations:** 1 Department of Cardiology, Hôpital du Sacré-Cœur de Montréal, University of Montreal, 5400 Gouin W Blvd, Montréal, QC H4J 1C5 Canada; 2 Division of Cardiology, Toronto General Hospital, University of Toronto, Toronto, ON Canada

**Keywords:** Three-dimensional echocardiography, Mitral valve, Mitral regurgitation

## Abstract

**Background:**

Mitral annulus (MA) area is derived during transthoracic echocardiography (TTE) assuming of a circular shape using the MA diameter from the apical 4 chamber (A4c) view. Since the MA is not a circular structure, we hypothesized that an elliptical model using parasternal long-axis (PLAX) and apical 2 chamber (A2c) view measured MA diameters would have better agreement with 3-dimensional transesophageal echocardiography (3D TEE) measured MA in degenerative mitral valve disease (DMVD).

**Methods:**

Seventy-six patients with moderate-to-severe DMVD had 2D TTE and 3D TEE performed. MA area was measured retrospectively using semi-automatic modeling of 3D data (3D TEE_sa_) and considered as the reference method. MA diameters were measured using different 2D TTE views. MA area was calculated using assumptions of a circular or an elliptical shape. 2D TTE derived and 3D TEE_sa_. MA areas were compared using linear regression and Bland-Altman analysis.

**Results:**

The median MA area measured at 3D TEE_sa_ was 1,386 (1,293–1,673) mm^2^. With 2D TTE, the circular model using A4c view diameter resulted in a small systematic underestimation of MA area (6%), while the elliptical model using PLAX and A2c diameters resulted in 25% systematic underestimation. The standard deviations of the distributions of inter-method differences were wide for all 2D TTE methods (265–289 mm^2^) when compared to 3D TEE_sa_, indicating imprecision.

**Conclusions:**

When compared with 3D TEE_sa_ modeling of the MA as the reference, the assumption of a circular shape using A4c TTE view diameter was the method with the least systematic error to assess MA area in DMVD and moderate to severe regurgitation.

**Supplementary Information:**

The online version contains supplementary material available at 10.1186/s44348-024-00001-w.

## Introduction

Degenerative mitral valve disease (DMVD) affects approximately 2% of the population and is the leading cause of mitral regurgitation (MR) in developed countries [1]. The primary modality for evaluation of the severity of MR is 2-dimensional transthoracic echocardiography (2D TTE) [2]. When qualitative and semi-quantitative parameters do not clearly establish the severity of the MR, quantitative methods are recommended [2]. One of them is the pulsed wave Doppler flow method (PWDF), which calculates the regurgitant volume (RVol) and fraction using pulsed wave Doppler combined with mitral annular (MA) and left ventricular outflow tract (LVOT) areas [2]. Precise assessment of MA area at 2D TTE is important to quantify the RVol and thus the severity of MR using the PWDF method [3]. The American Society of Echocardiography (ASE) recommends measuring MA diameter in the apical 4 chamber (A4c) view using the assumption of a circular shape to derive the area (0.785*diameter^2^) [2]. An alternative method is to use an assumption of an elliptical shape with the diameters measured in the A4c and apical 2 chamber (A2c) view [4, 5]. Recently, Hyodo et al. [6] have suggested that the best way to calculate the MA area with transesophageal echocardiography (TEE) was to use an assumption of an elliptical shape for the MA with the anteroposterior (AP) and intercommissural diameters. At TTE, the TEE AP diameter would correspond to a parasternal long-axis (PLAX) view and the TEE intercommissural diameter would be closer to an A2c view. However, there is concern that the standard TTE views do not measure the anatomically correct diameters [7].

In patients with severe DMVD and moderate to severe MR, the MA undergoes significant dilatation and remodeling [6, 8–10]. It is thus unclear whether the recommended formulas accurately assess the MA area from TTE annular diameters in this population. Furthermore, eligibility to the emergent transcatheter mitral valve (MV) procedures, including percutaneous annuloplasty, involves anatomical criteria such as the functional anatomy of MR and annular dimension provided by TTE. Recent guidelines recommend using a different approach to MA dimensions (apical LAX view and a modified A2c view) [11].

Three-dimensional transesophageal echocardiography allows precise semi-automated MV modeling (3D TEE_sa_) and measurements, including MA area obtained in the physiological state [12]. Assessment of MA area with 3D TEE_sa_ is a robust method and has shown excellent agreement with intraoperative measurements [10, 13, 14].

The main objective of this study was to compare the accuracy of 2D TTE (using different TTE views and geometric assumptions) to assess MA area in patients with DMVD and significant MR using 3D TEE_sa_ MA area measure as the reference standard. We hypothesized that the most accurate method to measure the MA area at 2D TTE would be to use the assumption of an elliptical shape using the MA diameters in the PLAX and A2c views.

## Methods

Adult subjects with moderate-to-severe degenerative MR who had diagnostic quality 2D TTE and 3D TEE at the Toronto General Hospital (*n* = 68), or at the Hôpital du Sacré-Coeur de Montréal (*n* = 8), Canada, were identified. The study protocol was approved by the Institutional Research Ethics Board at both institutions. Clinical data and grade of DMVD were gathered prospectively for all cases at the Toronto General Hospital. For patients of the Hôpital du Sacré-Coeur de Montréal, clinical data were obtained retrospectively in the medical files and the surgical grade of DMVD was not available.

### Echocardiograms

All TEE were performed on an iE33 or EPIQ 7 system (Philips Medical System, Andover, MA, USA) equipped with an X7-2t TEE transducer. TTE were performed on an iE33, EPIQ 7, or on a Vivid 7–9 system (GE Healthcare, Chicago, IL, USA). All studies were performed according to current guidelines [2, 15]. Traditional transthoracic echocardiographic imaging planes were obtained: PLAX, A2c, apical 3-chamber (A3c) and A4c views. The MA diameters were measured retrospectively in these imaging planes and a mid-systole frame was selected in order to have identical timing with 3D TEE measures.

3D TEE assessment of the MV was performed using full volume (median volume rate [VR], 20; vps [interquartile range; IQR, 16–26]) or real-time (median VR, 9; vps [IQR, 7–13]) 3D acquisitions of the MV from either the mid-esophageal 4- or 3-chamber view. Four beat gated acquisitions were used for the full-volume data sets. Withholding of respiration was performed whenever possible.

Fifty-four (71%) patients had both TTE and the 3D TEE performed the same day. For the remaining patients, the median time period between the TTE and the 3D TEE was 55 days (28–125).

### 3D quantitative measurements

**Fig. 1:**
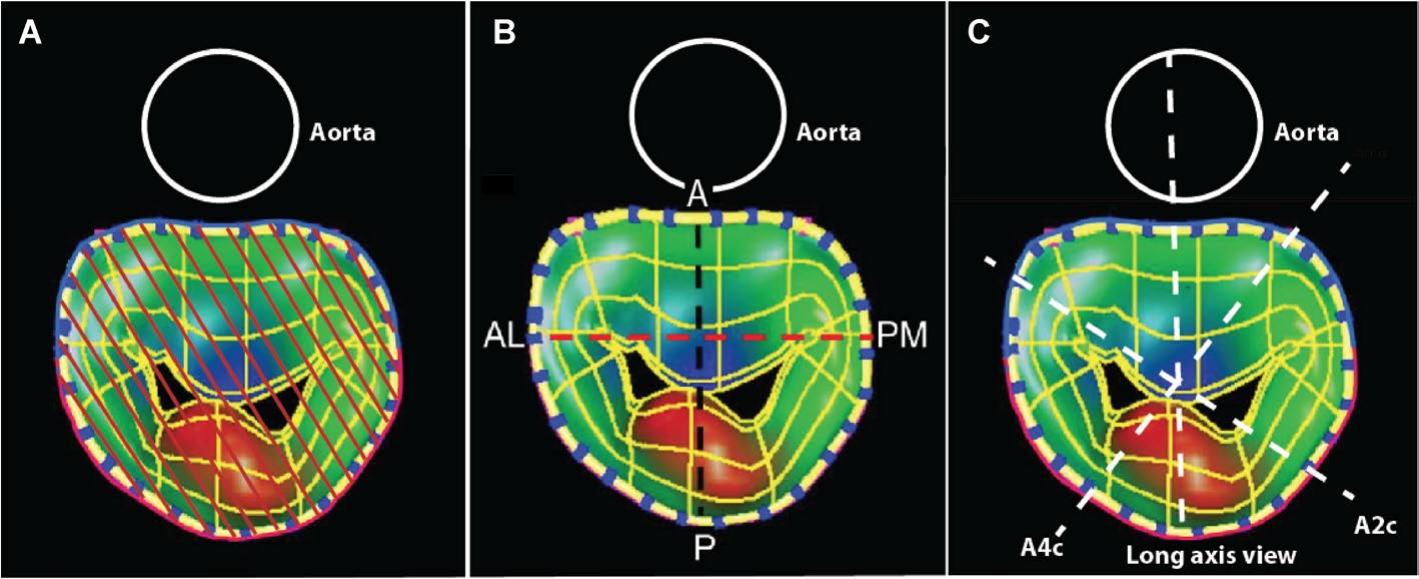
Quantitative parameters of MA. 3-dimensional shape of the MA automatically generated from a geometric model (**A**, **B**). MA area (3D TEE_sa_ MA area) (**A**), and anatomically correct 3D TEE AP and ALPM diameters (**B**) were derived. Approximate position of cut planes obtained with standard transthoracic echocardiographic views (**C**). A4c: apical 4 chamber, ALPM: anterolateral to posteromedian, AP: anteroposterior, A2c: apical 2 chamber, MA: mitral annulus, 3D TEE: 3-dimensional transesophageal echocardiography, 3D TEE_sa_: 3-dimensional transesophageal echocardiography semi-automated method, long-axis view: either parasternal long axis view or apical 3 chamber view

For exams performed at the Toronto General Hospital (*n* = 68), the 3D TEE data was gathered prospectively as a part of a previously published study [12]. The 3D TEE data were analyzed retrospectively for exams performed at the Hôpital du Sacré-Coeur de Montréal (*n* = 8). The 3D TEE datasets were first assessed for gating artifacts by examining the studies in a plane perpendicular to the plane of acquisition. Studies with gating artifacts were excluded. The studies were then analyzed offline by 2 operators blinded to clinical and echocardiographic findings. A mid-systolic frame was chosen for analysis. MA area was measured using 3D TEE_sa_ (eSie Valves, Siemens, Mountain View, CA, USA) (Fig. 1A), which has been previously described in details [12, 16]. The anatomically correct MA diameters (AP and anterolateral to posteromedian [ALPM]) were also measured from the 3D semi-automated mitral valve modeling (Fig. 1B).

### Comparison of 2D TTE and 3D TEE_sa_ MA area

We compared the MA area derived from 2D TTE diameters with the MA area directly measured using 3D TEE_sa_. The 2D TTE derived MA area was calculated with different methods [2, 4, 5]:

- Method 1: assumption of a circular shape for the MA. Measure of MA diameter in A4c view (d).
  $$\mathrm{MA}\;\mathrm{area}=3.14\;(\text{d}/2)^2$$
- Method 2: assumption of an elliptical shape for the MA. Measure of MA diameters in A2c (d1) and A4c views (d2).
  $$\mathrm{MA}\;\mathrm{area}=3.14\;(\text{d}1\ast\text{d}2)/4$$
- Method 3 (study hypothesis): Assumption of an elliptical shape for the MA. Measure of MA diameters in PLAX (d1) and A2c (d2) views.
  $$\mathrm{MA}\;\mathrm{area}=3.14\;(\text{d}1\ast\text{d}2)/4$$

### Exploratory analysis

#### Validation of MA diameters and geometric models

We wanted to assess whether the assumption of an elliptical shape using measurements that are obtained in an anatomically correct way using multiplanar reconstructions of the 3D mitral annulus would provide a better estimate of the 3D MV area than the assumption of a circular shape. For each patient, MA area was derived from 3D-TEE anatomically correct diameters using 3 geometric models: (1) a circular model [3.14 (d/2)^2^] with the AP diameter, (2) a circular model with the ALPM diameter, and (3) an elliptical model [3.14 (d1*d2)/4] with both diameters. These derived MA areas were then compared to the MA area directly measured with the semi-automated software (3D TEE_sa_ MA area).

#### Comparison of 3D TEE and 2D TTE diameters

We also assessed whether MA diameters measured at 2D TTE were representative of anatomically correct measurements. To do so, we compared diameters measured at 2D TTE with the anatomically correct diameters measured at 3D TEE.

### Intra- and inter-observer variability

Ten randomly selected exams were re-analyzed by the same operator 3–5 months after the initial measure in order to determine intra-observer variability of each MV annular area method and 2D TTE MA diameters. A second operator, blinded to results and to the precise frame used, reanalysed 10 2D TTE and 3D TEE datasets in order to determine inter-observer variability. This analysis was done for 2D TTE diameters, 2D TTE-derived MA areas, 3D TEE diameters and 3D TEE_sa_ MA area.

### Statistical analysis

Categorical variables are expressed in frequency and percentages. Normality of distribution of continuous variables are assessed with the Shapiro-Wilk test. Continuous variables are described as mean ± standard deviation (SD) if the distribution was normal, otherwise as median and IQR (25th–75th). Each MA area derived using 2D TTE or 3D TEE diameters was compared with the 3D TEE_sa_ measured MA area using linear regression and Bland-Altman analysis. Diameters measured at 2D TTE were compared with those measured with the 3D TEE with the same analysis.

Using Bland–Altman analysis, for each method tested, we evaluated systematic bias (using mean or median of differences between methods), SD of inter-method difference, precision (range within which are 95% of values of differences between methods, i.e. ± 1.96 SD or 2.5th–97.5th percentile of differences between methods) and percent error [precision/median value of 3D TEE_sa_ MA area (the median 3D TEE_sa_ MA area was used since its distribution was non-normal)] [17]. The distributions of the inter-methods differences were tested for normality with the Shapiro-Wilk test with a threshold value of < 0.05. If the distribution of differences between methods was non-normal, we used the median and 2.5th–97.5th of difference between methods to evaluate respectively systematic bias and precision. The intra- and inter-observer variability were evaluated with the intraclass correlation coefficient and the coefficient of variation (for each 2D method and for the 3D TEE method). Analyses were performed with the use of JMP version 12.0.1 (SAS Institute Inc., Cary, NC, USA) and MedCalc version 16.8.4 (MedCalc Software, Ostend, Belgium). When applicable, a 2-tailed p-value of 0.05 was used for all analyses.

## Results

Seventy-six patients (59 ± 10 years, 70% male) with DMVD and moderate to severe MR were included, of which 75 (99%) had surgical MV repair (Table 1). Amongst them, 62% had ≥ New York Heart Assessment class 2 symptoms at the time of surgery, with normal left ventricular (LV) size (end-systolic diameter < 40 mm) in 87% and normal LV ejection function (≥ 60%) in 57%. Data on the surgical inspection of the mitral valve was available for 68 patients (89%). Of these, 21 (31%) had minimal myxomatous changes indicating fibroelastic deficiency while 47 (69%) had moderate or severe myxomatous changes (Supplementary Table 1). The baseline MA measures at 2D TTE and 3D TEE are shown in Table 2. The 2D TTE A2c and A4c views diameters were similar (mean value of 40 and 42 mm, respectively) and larger than the A3c and PLAX views diameters (both 35 mm). At 3D TEE, the mean AP diameter was 37 ± 5 mm and the median ALPM diameter was 44 mm (41–48 mm). The median MA area at 3D TEE_sa_ was 1,386 mm^2^ (1,293–1,673 mm^2^).

**Table 1:** Baseline characteristics

Baseline characteristics	Total (*n* = 76)
Age (years)	59 ± 10
Male	53 (70)
BSA (m^2^)	1.9 ± 0.2
Hypertension (*n* = 74)	23 (31)
Diabetes (*n* = 75)	4 (5)
AF (*n* = 74)^a^	13 (18)
NYHA class	2 (1–2)
2D Echocardiography	
LV end-diastolic diameter (mm)	52 (49–55)
LV end-systolic diameter (mm)	33 (28–36)
LVEF (%)	61 ± 8
RVSP (mmHg)	31 (26–43)

Values are mean ± standard deviation or median (interquartile range), and frequencies (percentage)

*AF* atrial fibrillation, *BSA* body area area, *LV* left ventricular, *LVEF* left ventricular ejection fraction, *NYHA* New York Heart Assessment, *RVSP* right ventricular systolic pressure, *3D TEE* 3-dimensional transesophageal echocardiography, *2D TTE* 2-dimensional transesophageal echocardiography

^a^The 13 patients were in AF during both 3D TEE and 2D TTE

**Table 2:** MA measures at 2D TTE and 3D TEE in the whole cohort

MA measures	Whole cohort (*n* = 76)
2D TTE MA measures	
A4c diameter (mm)	42 (39–45)
A3c diameter (mm)	35 (33–38)
A2c diameter (mm)	40 ± 5
PLAX diameter (mm)	35 (33–39)
Method 1, MA area (mm^2^) (circular assumption, A4c)	1,382 (1,182–1,581)
Method 2, MA area (mm^2^) (elliptical assumption, A2c-A4c)	1,275 (1,118–1,493)
Method 3, MA area (mm^2^) (elliptical assumption, A2c-PLAX)	1,082 (951–1,241)
3D TEE MA measures	
AP diameter (mm)	37 ± 5
ALPM diameter (mm)	44 (41–48)
AP/ALPM diameters ratio	0.83 ± 0.09
MA area, direct measurement by semi-automated method (mm^2^)	1,386 (1,293–1,673)

*A4c* apical 4 chamber, *ALPM* anterolateral to posteromedian, *AP* anteroposterior, *A3c* apical 3 chamber, *A2c* apical 2 chamber, *MA* mitral annulus, *PLAX* parasternal long axis, *3D TEE* 3-dimensional transesophageal echocardiography, *2D TTE* 2-dimensional transesophageal echocardiography

### Comparison of 2D TTE methods with 3D TEE_sa_ MA area

**Fig. 2:**
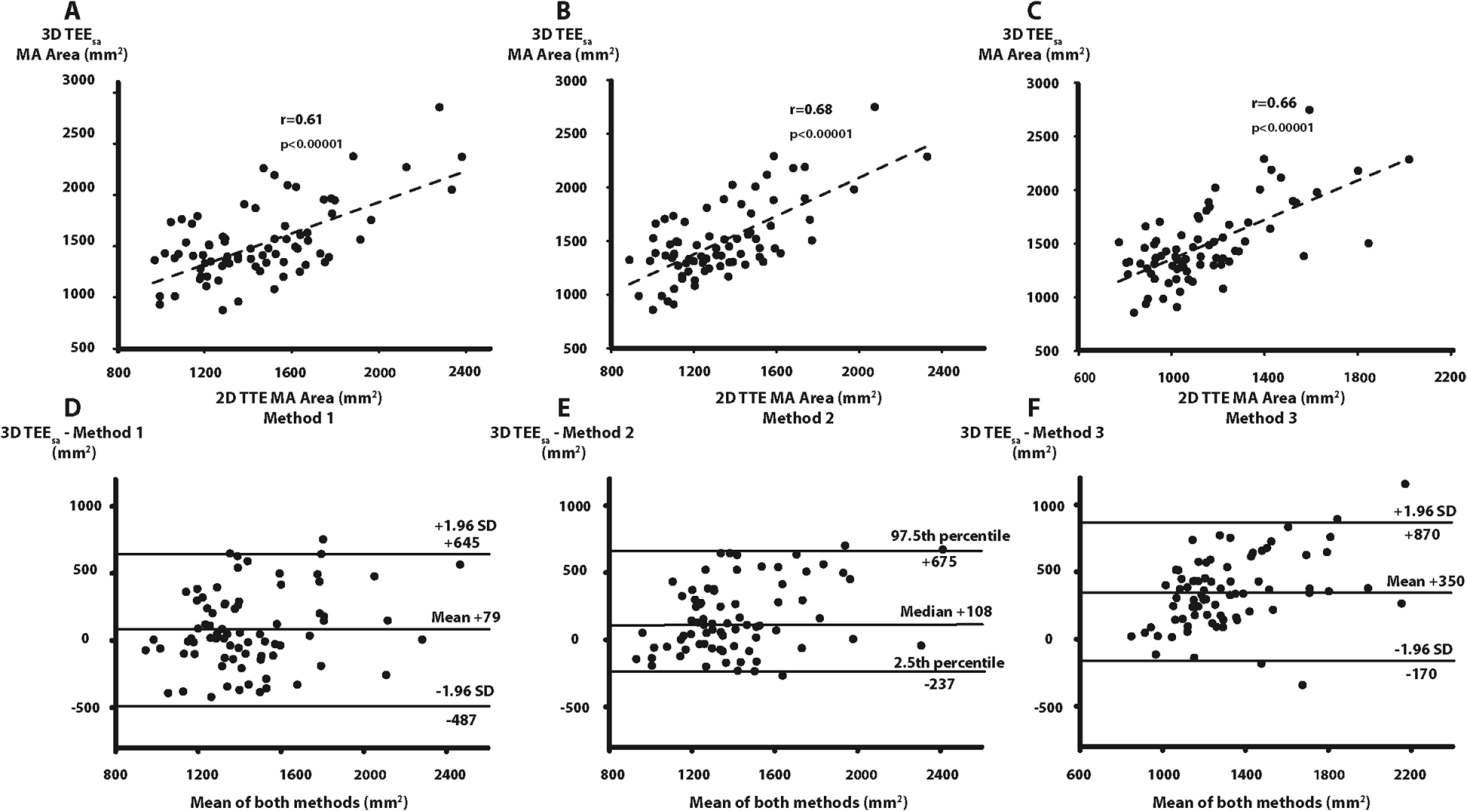
Comparison between 3D TEE semi-automatic MA area and MA area derived from 2D TTE MA diameters. Linear regression analysis between 3D TEE_sa_ MA area and 2D TTE area by method 1 (**A**), linear regression analysis between 3D TEE_sa_ MA area and 2D TTE area by method 2 (**B**), linear regression analysis between 3D TEE_sa_ MA area and 2D TTE area by method 3 (**C**), Bland-Altman analysis of 3D TEE_sa_ MA area and 2D TTE MA area by method 1 (**D**), Bland-Altman analysis of 3D TEE_sa_ MA area and 2D TTE MA area by method 2 (**E**), Bland-Altman analysis of 3D TEE_sa_ MA area and 2D TTE MA area by method 3 (**F**). Method 1: assumption of a circular shape for the MA. Measure of MA diameter in apical 4 chamber (d). MA area = 3.14 (d/2)^2^. Method 2: assumption of an elliptical shape for the MA. Measure of MA diameters in apical 2 (d1) and 4 chambers (d2). MA area = 3.14 (d1*d2)/4. Method 3: assumption of an elliptical shape for the MA. Measure of MA diameters in PLAX (d1) and apical 2 chambers (d2). MA area = 3.14 (d1*d2)/4. MA: mitral annulus, SD: standard deviations, 3D TEE: 3-dimensional transesophageal echocardiography, 3D TEE_sa_: 3-dimensional transesophageal echocardiography semi-automated method, 2D TTE: 2-dimensional transthoracic echocardiography

The correlations between MA areas derived from 2D TTE MA diameters and 3D TEE_sa_ MA area were all modest (r between 0.60 and 0.68, Fig. 2A and C).

#### Analysis of systematic bias

At Bland-Altman analysis, all 2D methods showed statistically significant systematic underestimation of MA area compared with 3D TEE_sa_ MA area (Table 3, Fig. 2D to (and) F). Method 1 (MA diameter in apical 4c with the assumption of a circular shape for the MA, as recommended in the 2017 ASE guidelines) and method 2 (MA diameters measured in A2c and A4c views with the assumption of an elliptical shape for the MA), showed small systematic underestimations of 6% and 8%, respectively, whereas method 3 (assumption of an elliptical shape for the MA using diameters in PLAX and A2c views) showed systematic underestimation of 25%. An alternative elliptical model using PLAX and A4c diameters showed systematic underestimation of 21% (Supplementary Table 2).

**Table 3:** Comparison between 3D TEE semi-automatic MA area and MA area derived from 2D TTE MA diameters

MA area derived from 2D TTE MA diameters	Mean difference between methods ± SD or median difference (25th–75th percentiles), (mm^2^)	% of systematic underestimation	Precision (mm^2^)	Percent error (%)
Method 1, MA area (mm^2^) (circular model, A4c)	79 ± 289	6	1,132	82
Method 2, MA area, (mm^2^) (elliptical model, A2c-A4c)	108 (− 51 to + 370)	8	912	66
Method 3, MA area, (mm^2^) (elliptical model, A2c-PLAX)	350 ± 265	25	1,040	75

*A4c* apical 4 chamber, *A3c* apical 3 chamber, *A2c* apical 2 chamber, *MA* mitral annulus, *PLAX* parasternal long-axis, *SD* standard deviations, *3D TEE* 3-dimensional transesophageal echocardiography, *2D TTE* 2-dimensional transesophageal echocardiography

Severe degree of degenerative myxomatous changes (*n* = 15) was associated with increased bias of underestimation (method 1, 19%; method 2, 16%; and method 3, 33%).

#### Analysis of precision

The SDs of the distribution of inter-method differences between 2D TTE and 3D TEE_sa_ MA area were large for all 2D TTE methods. The SD was of ± 289 mm^2^ (± 21% of the median 3D TEE_sa_ MA area) for method 1, the 25th to 75th interquartile was (− 51 to + 370) for method 2 (non-standard distribution) and the SD was ± 265mm^2^ (± 19% of the median 3D TEE_sa_ MA area) for method 3 (Table 3, Fig. 2D and F). Consequently, the 95% confidence intervals (CIs) of inter-method differences (± 1.96 SD or 2.5th to 97.5th interquartile, i.e. precision) were also large: 1,132 mm^2^ for method 1, 912 mm^2^ for method 2 and 1,040 mm^2^ for method 3. The percent error (95% CI of inter-method differences/median value of 3D TEE_sa_ MA area) was 82% for method 1, 66% for method 2 and 75% for method 3 (Table 3).

Other 2D TTE circular of elliptical models using diameters from alternative TTE views or combinations of TTE views were tested, with similar results (Supplementary Table 2).

### Exploratory analysis

#### Validation of a geometric model at 3D TEE

**Fig. 3:**
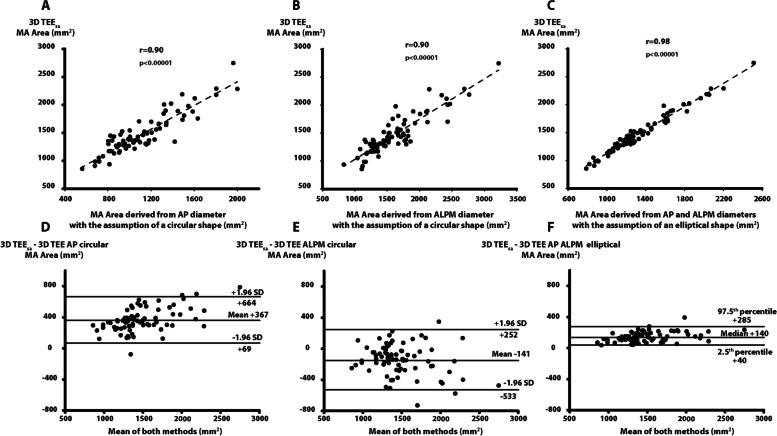
Comparison between 3D TEE semi-automated MA area direct measurement and MA area derived from 3D TEE anatomically correct diameters. Linear regression analysis between 3D TEE_sa_ MA area direct measurement and MA area derived from 3D TEE AP diameter using an assumption of a circular shape for the MA (**A**). Linear regression analysis between 3D TEE_sa_ MA area direct measurement and MA area derived from 3D TEE ALPM diameter using an assumption of a circular shape (**B**). Linear regression analysis between 3D TEE_sa_ MA area direct measurement and MA area derived from 3D TEE AP and ALPM diameters using an assumption of an elliptical shape for the MA (**C**), Bland-Altman analysis of 3D TEE_sa_ MA area direct measurement and MA area derived from 3D TEE AP diameter using a circular assumption for the MA (**D**), Bland-Altman analysis of 3D TEE_sa_ MA area direct measurement and MA area derived from 3D TEE ALPM diameter using an assumption of a circular shape for the MA (**E**), Bland-Altman analysis of 3D TEE_sa_ MA area direct measurement and MA area derived from 3D TEE AP and ALPM diameters using an assumption of an elliptical shape for the MA (**F**). ALPM: anterolateral to posteromedian, AP: anteroposterior, MA: mitral annulus, 3D TEE: 3-dimensional transesophageal echocardiography, 3D TEE_sa_: 3-dimensional transesophageal echocardiography semi-automated method

All MA areas derived from 3D TEE anatomically correct diameters showed excellent correlation with 3D TEE_sa_ MA area direct measurement (r between 0.9 and 0.98) (Fig. 3A and C). The best geometrical model was the elliptical model (AP and ALPM diameters) with a very strong correlation of 0.98, a systematic underestimation of 10% and good precision (95% CIs of inter-method differences 245 mm^2^, percent error of 18%) (Fig. 3D and F, Supplementary Table 3). The AP circular model showed systematic underestimation 26% while the ALPM circular model showed a systematic overestimation of 10%. Both circular models showed inferior precision compared with the elliptical model (596 and 784 mm^2^, percent error 43 and 57% for AP and ALPM circular models, respectively).

#### Comparison of 3D TEE and 2D TTE diameters

**Fig. 4:**
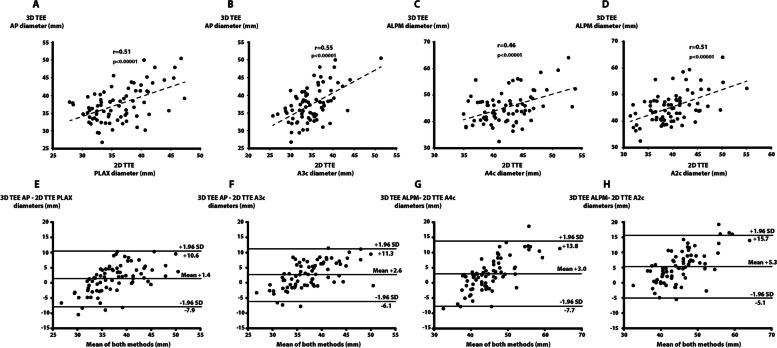
Comparison between 3D TEE and 2D TTE diameters. Linear regression analysis between 3D TEE AP diameter and 2D TTE PLAX diameter (**A**), linear regression analysis between 3D TEE AP diameter and 2D TTE A3c diameter (**B**), linear regression analysis between 3D TEE ALPM diameter and 2D TTE A4c diameter (**C**), linear regression analysis between 3D TEE ALPM diameter and 2D TTE A2c diameter (**D**), Bland-Altman analysis of 3D TEE AP diameter and 2D TTE PLAX diameter (**E**), Bland-Altman analysis of 3D TEE AP diameter and 2D TTE A3c diameter (**F**), Bland-Altman analysis of 3D TEE ALPM diameter and 2D TTE A4c diameter (**G**), Bland-Altman analysis of 3D TEE ALPM diameter and 2D TTE A2c diameter (**H**). ALPM: anterolateral to posteromedian, AP: anteroposterior, A4c: apical 4 chamber, A3c: apical 3 chamber, A2c: apical 2 chamber, PLAX: parasternal long-axis, 3D TEE: 3-dimensional transesophageal echocardiography, 2D TTE: 2-dimensional transthoracic echocardiography

The correlations between 3D TEE AP and corresponding 2D TTE apical 3c or PLAX diameters were both weak (r of respectively 0.55 and 0.51) (Fig. 4A and D). The correlations between the 3D TEE ALPM diameter and the 2D TTE apical 4c or apical 2c were also poor (r of respectively 0.46 and 0.51). The 2D TTE A2c view underestimated the 3D TEE ALPM diameter by 5.3 mm (12% systematic underestimation), while the 2D TTE A4c view underestimated the 3D TEE ALPM diameter by 3.0 mm (7% systematic underestimation) (Fig. 4 E and F, Supplementary Table 4). The 2D TTE PLAX diameter slightly underestimated the 3D TEE AP diameter by 1.4 mm (4% systematic underestimation) and the 2D TTE A3c diameter underestimated the 3D TEE AP diameter by 2.6 mm (7% systematic underestimation).

For all 2D TTE diameters measurements, there was lack of precision when compared to 3D TEE diameters measures (percent error between 46 and 49%) (Fig. 4 E and F, Supplementary Table 4).

### Reproducibility

Analysis of inter- and intra-observer variability for the mitral valve measurements obtained demonstrated good agreement between observations (Supplementary Table 5).

## Discussion

An assumption of a circular shape is used to measure the MA area for quantification of MR at TTE. However, this assumption is not anatomically correct, as the MA is a complex 3-dimensional D-shaped structure, with a saddle shape that would be closer to an ellipse than to a circle [18, 19]. In a recent study of patients with various cardiac conditions, the MA area derived from anatomically correct diameters using an assumption of an elliptical shape at TEE was better than circular models when compared with 3D MA planimetry [6]. We confirmed these findings in our explanatory analysis: when using anatomically correct MA diameters obtained from multiplanar reformats of 3D TEE data, the elliptical model was superior to circular models at 3D TEE and resulted in a better correlation with 3D TEE_sa_ MA area. However, quantification of MR is done at TTE, in which the measured MA diameters are not anatomically correct. Thus, it was unclear whether an assumption of an elliptical shape would be superior to a circular model at TTE.

In this study of patients with DMVD and moderate-severe MR, we aimed to find whether the use of an assumption of an elliptical shape with MA diameters measured at TTE would increase the accuracy of the MA area measure compared to circular geometric models. The main results were: (1) MA diameters measured at 2D TTE showed poor correlation with 3D TEE diameters; (2) The use of an assumption of an elliptical shape for the MA at 2D TTE using PLAX and A2c (or A4c) diameters resulted in large underestimation (25% and 21%, respectively) of the MA area compared with 3D TEE_sa_, without increasing precision, thus invalidating the study hypothesis. An assumption of a circular shape using the A4c view diameter or an assumption of an elliptical shape using the A2c and A4c views diameters resulted in small systematic underestimations (respectively 6% and 8%) of the MA area by 3D TEE_sa_. (3) All geometric assumptions that used 2D TTE diameters for MA area calculation were imprecise compared with 3D TEE_sa_.

MA diameters measured at 2D TTE are not anatomically correct. Indeed, there was systematic underestimation between the diameters measured at 2D TTE and 3D TEE, particularly for the measure of the ALPM diameter, which corresponded neither to A2c nor to A4c TTE diameter (Fig. 1C). The use of an assumption of an elliptical shape for the MA at 2D TTE using PLAX view MA diameter (small systematic underestimation compared with AP true anatomical diameter) and either A2c or A4c view MA diameter (larger systematic underestimation compared with ALPM true anatomical diameter) resulted in severe underestimation of the MA area while the assumption of a circular shape using the A4c view diameter or the elliptical model using A4 and A2c resulted in higher MA value, closer to the 3D TEE values.

Moreover, there was imprecision of MA diameters measurements at 2D TTE when compared to 3D TEE, translating into a lack of precision of 2D TTE geometric models derived MA area compared with 3D TEE_sa_.

These results are consistent with the study of Foster et al. [7] that showed poor correlation between MA diameters measured using traditional TTE views (A4c view and PLAX view) and MA diameters measured at cardiac CT. In that study, the use of anatomically optimized TTE views allowed the measurement of anatomically correct diameters (AP and ALPM diameters, Fig. 1B). The described method is the following: in a first step, the correct imaging plane of the ALPM axis is localized when P3, A2, and P1 are seen equally in the same imaging plane (similarly to a commissural view at TEE). Then, rotation of the imaging plane 90 degrees allows measurement of the AP axis. The anterior border of the MA is measured at the junction of the left atrium and leaflet [7]. That method increased significantly the correlation with diameters measured at cardiac CT. However, this was a small study (17 patients) and there is actually no recommendation to use anatomically optimized views to measure the MA [2]. The apical 2-chambes view seem particularly prone to variability in the anatomic plane shown and how it transects the LV and the MV. For instance, an A2C view showing 2 mitral leaflets (A2-P2) transects the anterior and the junction of the inferior and posterior LV walls, while clockwise probe rotation produces an optimal A2C with 3 mitral scallops (P3-A2-P1) transecting the inferior and antero-lateral LV walls.

PWDF method to assess Rvol was initially validated against thermodilution and thereafter as a severity parameter in large outcomes studies of patients with primary or functional MR [4, 20–23]. However, it has limitations. In a recent study which compared different methods to calculate the RVol, the PWDF method showed imprecision and systematic error in comparison to cardiac magnetic resonance (*r* = 0.56; mean overestimation of MR volume of + 51 mL, 1.96 SD inter-method difference in MR volume 94.2 mL) [24]. This could be due in part to the suboptimal precision of the measure of MA diameters with traditional imaging planes, thus reinforcing the hypothesis that the use of anatomically optimized views as described by Foster et al. [7] could increase the precision of that method.

Moreover, in DMVD of increasing complexity, the annulus undergoes significant dilatation particularly along the AP axis, creating a specific pattern of enhanced circularity with a reduced ratio of commissural diameter to AP diameter compared to its normal saddle-shape [9, 10].

As explained by Grewal et al. [10], during systole, DMVD annulus contracts anteroposteriorly but less than normal and is compensated for by persistent intercommissural widening, leading to absent overall annular area contraction. This contrasts with ischemic MR annulus, which, despite similar AP enlargement, is narrower and essentially adynamic [10]. That might explain our finding that the circular equation based on the largest annular diameter (A4c) approximated better the MV annular area and that severe degree of degenerative myxomatous changes was associated with increased underestimation of the study hypothesis elliptical model (33%).

Novel 3D echocardiographic quantitative methods are also promising for improving quantification of MR. New 3D recommended methods include the measure of the vena contracta area or the calculation of total stroke volume by measuring the ventricular volumes (in diastole and systole) [2]. 3D automated stroke volume measurement, automated real-time 3D volume color flow Doppler proximal isovelocity surface area measure and hybrid imaging (assessment of MA area at 3D echocardiography combined with 2D TTE pulsed wave Doppler) are also promising [24–26]. The use of 3D echocardiography measured MA area (and optimised diameters using 3D guided biplane imaging) and LVOT area combined with pulsed wave Doppler techniques may be a better method to quantify MR. These methods will need clinical validation.

Finally, with the development of alternative novel transcatheter therapies for MR, some of which targeting annular repair with direct or indirect annuloplasty, precise assessment of MA remodeling is essential [11]. In DMVD specifically, our work favors the use of a simpler circular model using the MA diameter in the A4c view.

The main technical limitation of this study is the fact that the measures were done in systole. A mid-systolic frame was chosen because it was where leaflet billowing and/or prolapse was best visualized to ensure optimal visualization for robust modeling. Moreover, we believe that the choice of a mid-systolic frame is interesting because it captures the unique alterations in MA dynamics seen specifically in DMVD and occurring in systole. In addition, this was a retrospective study that used traditional TTE views that were not optimized to be truly anatomical, which reflects the reality of clinical practice. Current ASE guidelines do not recommend using anatomically optimized views for the assessment of MA area [2]. Despite these limitations, this study remains the largest study comparing the measurement of MA by 2D TTE to 3D-TEE semi-automated method in patients with severe DMVD. Finally, cardiac computed tomography which has become the reference method rather than 3D TEE for pre-procedural assessment and quantification of the mitral valve was not available [27].

In this study of patients with moderate to severe degenerative MR, the assumption of a circular shape for the MA using A4c TTE diameter was the method with the least systematic error (6% underestimation) when compared with 3D TEE_sa_ MA area. An assumption of an elliptical shape using MA diameters measured in PLAX and A2c TTE views was not superior to the assumption of a circular shape using A4c MA diameter as it resulted in large underestimation of MA area. All 2D TTE measurements showed imprecision when compared to 3D TEE, thus more accurate methods for the assessment of MA diameters and area should be investigated and the effect on the grading of the severity of MR.

### Supplementary Information

**Additional file 1:** **Supplementary Table 1. **Grade of DVMD at surgical inspection. **Supplementary Table 2. **Comparison between 3D TEE semi-automatic MA area and MA area derived from 2D TTE MA diameters with circular or elliptical assumptions using other combinations of diameters. **Supplementary Table 3. **Comparison between 3D TEE semi-automated direct MA area measure and MA area derived from 3D TEE anatomically correct diameters. **Supplementary Table 4. **Comparison between 3D TEE and 2D TTE diameters. **Supplementary Table 5.  **Reproducibility analysis (*n* = 10): ICC and coefficient of variation.
