# Supplementary material for: Two-dimensional transthoracic measure of mitral annulus in mitral valve prolapse and moderate to severe regurgitation: a method comparison analysis with three-dimensional transesophageal echocardiography
Source: J Cardiovasc Imaging. 2024 Jun 12;32:2. doi: 10.1186/s44348-024-00001-w (PMC11177645; doi:10.1186/s44348-024-00001-w)
Supplement: Supplementary file 1 — Additional file 1: Supplementary Table 1. Grade of DVMD at surgical inspection. Supplementary Table 2. Comparison between 3D TEE semi-automatic MA area and MA area derived from 2D TTE MA diameters with circular or elliptical assumptions using other combinations of diameters. Supplementary Table 3. Comparison between 3D TEE semi-automated direct MA area measure and MA area derived from 3D TEE anatomically correct diameters. Supplementary Table 4. Comparison between 3D TEE and 2D TTE diameters. Supplementary Table 5. Reproducibility analysis (n = 10): ICC and coefficient of variation. [file 44348_2024_1_MOESM1_ESM.docx]

**Online Supplementary Materials**

**for the following Article:**

Two-dimensional transthoracic measure of mitral annulus in mitral valve prolapse and moderate to severe regurgitation. A method comparison analysis with three-dimensional transesophageal echocardiography (JCVI-23-009)

Maxime Berthelot-Richer MD MSc ^a^, Halyna Viktorivna Vakulenko MD ^a^, Anna Calleja MD ^b^, Anna Woo ^b^ MD SM, Paaladinesh Thavendiranathan MD MSc ^b^ and Frédéric Poulin MD MSc ^a^

**Table S1**

**Grade of DVMD at surgical inspection**

|  | **Patients with available data**  **n=68** |
| --- | --- |
| **Myxomatous change, n (%)** |  |
| **Mild** | **21 (31)** |
| **Moderate** | **32 (47)** |
| **Severe** | **15 (22)** |

Grading of DMVD was based on the intraoperative leaflet and annular changes seen during surgical inspection. Mild myxomatous degeneration was defined as leaflets that were thin, fairly normal in size, transparent (except for the prolapsing segment), and had chordae tendineae that were thin and attenuated. This includes Carpentier fibroelastic deficiency and cases with minimal myxomatous changes.  Moderate was defined as opaque leaflets due to myxoid infiltration of the spongiosa, increased leaflet size but still elastic and not excessively thick (<3mm thickness), and the chordae tendineae had myxoid infiltration. Severe was when leaflets were voluminous, aneurysmal, and thickened (≥3mm), massively dilated annulus (ie, ≥40mm) and often posterior displacement of the mitral annulus ≥5mm, with thick and obviously myxomatous chordae tendineae.

**Table S2**

**Comparison between 3D TEE semi-automatic MA area and MA area derived from 2D TTE MA diameters with circular or elliptical assumptions using other combinations of diameters**

|  | **Mean difference between methods ± SD, mm^2^** | **% of systematic underestimation** | **Precision, mm^2^** | **Percent error, %** |
| --- | --- | --- | --- | --- |
| Circular assumption, A2c | 224 ± 292 | 16 | 1145 | 83 |
| Elliptical assumption, A2c-A3c | 389 ± 285 | 28 | 1117 | 81 |
| Elliptical assumption, PLAX-A4c | 287 ± 259 | 21 | 1015 | 73 |
| Elliptical assumption, A3c-A4c | 329 ± 278 | 24 | 1090 | 79 |

A4c= apical 4 chamber view; A2c= apical 2 chamber view; A3c= apical 3 chamber view; PLAX= parasternal long-axis view; MA= mitral annulus

**Table S3**

**Comparison between 3D TEE semi-automated direct MA area measure and MA area derived from 3D TEE anatomically correct diameters**

|  | **Mean difference between methods ± SD, or median difference [25th-75^th^ interquartiles], mm^2^** | **% of systematic error** | **Precision, mm^2^** | **Percent error, %** |
| --- | --- | --- | --- | --- |
| Circular assumption, AP diameter | 367 ± 152 | -26 | 596 | 43 |
| Circular assumption, ALPM diameter | -141 ± 200 | +10 | 784 | 57 |
| Elliptical assumption, combination of AP and ALPM diameters | 140 [101-190] | -10 | 245 | 18 |

AP= anteroposterior; ALPM= anterolateral to posteromedian; 3D TEE = three-dimensional transesophageal echocardiography; 2D TTE= two-dimensional transthoracic echocardiography; MA= mitral annulus

**Table S4. Comparison between 3D TEE and 2D TTE diameters**

|  | **Mean difference between methods ± SD, mm** | **% of systematic underestimation** | **Precision, mm** | **Percent error, %** |
| --- | --- | --- | --- | --- |
| **Comparison with 3D TEE AP diameter** | | | | |
| 2D TTE PLAX | 1.4 ± 4.7 | 4 | 18 | 49 |
| 2D TTE A3c | 2.6 ± 4.4 | 7 | 17 | 46 |
| **Comparison with 3D TEE ALPM diameter** | | | | |
| 2D TTE A4C | 3.0 ± 5.5 | 7 | 22 | 49 |
| 2D TTE A2c | 5.3 ± 5.3 | 12 | 21 | 47 |

A4c= apical 4 chamber view; A2c= apical 2 chamber view; A3c= apical 3 chamber view; PLAX= parasternal long-axis view; AP= anteroposterior; ALPM= anterolateral to posteromedian; 3D TEE = three-dimensional transesophageal echocardiography; 2D TTE= two-dimensional transthoracic echocardiography

| **Table S5: Reproducibility analysis (n=10): ICC and coefficient of variation** | | | | |
| --- | --- | --- | --- | --- |
|  | **Intra-observer variability** | | **Inter-observer variability** | |
|  | ICC | Coefficient of variation (%) | ICC | Coefficient of variation (%) |
| **3D TEE_sa_ MA area** | 0.99 | 1.0 | 0.99 | 1.7 |
| **3D TEE MA Diameters** |  |  |  |  |
| Anteroposterior  ALPM | 0.99  0.97 | 1.6  1.3 | 0.99  0.99 | 2.9  1.9 |
| **2D TTE MA Diameters** |  |  |  |  |
| Parasternal long-axis | 0.98 | 1.4 | 0.91 | 5.6 |
| Apical 4-chamber | 0.99 | 0.9 | 0.87 | 3.8 |
| Apical 2-chamber | 0.99 | 0.6 | 0.73 | 5.6 |
| Apical 3-chamber | 0.98 | 1.0 | 0.94 | 2.4 |
| **2D TTE-derived MA areas** |  |  |  |  |
| Method 1 | 0.99 | 1.7 | 0.89 | 7.6 |
| Method 2 | 0.99 | 1.2 | 0.83 | 8.4 |
| Method 3 | 0.99 | 1.7 | 0.91 | 10.5 |
| 3D TEE= three-dimensional transesophageal echocardiography; 2D TTE= two-dimensional transthoracic echocardiography; ALPM= anterolateral to posteromedian; ICC= Intraclass correlation coefficient; MA= mitral annulus; | | | | |
